# Total joint arthroplasty: practice variation of physiotherapy across the continuum of care in Alberta

**DOI:** 10.1186/s12913-016-1873-9

**Published:** 2016-11-04

**Authors:** C. Allyson Jones, Ruben San Martin, Marie D. Westby, Lauren A. Beaupre

**Affiliations:** 1 Department of Physical Therapy, Faculty of Rehabilitation Medicine, University of Alberta, Edmonton, AB T6G 2G4 Canada; 2 School of Public Health, University of Alberta, Edmonton, Alberta T6G 1C9 Canada

**Keywords:** Hip arthroplasty, Knee arthroplasty, Physical therapy, Practice, Rehabilitation

## Abstract

**Background:**

Comprehensive and timely rehabilitation for total joint arthroplasty (TJA) is needed to maximize recovery from this elective surgical procedure for hip and knee arthritis. Administrative data do not capture the variation of treatment for rehabilitation across the continuum of care for TJA, so we conducted a survey for physiotherapists to report practice for TJA across the continuum of care. The primary objective was to describe the reported practice of physiotherapy for TJA across the continuum of care within the context of a provincial TJA clinical pathway and highlight possible gaps in care.

**Method:**

A cross-sectional on-line survey was accessible to licensed physiotherapists in Alberta, Canada for 11 weeks. Physiotherapists who treated at least five patients with TJA annually were asked to complete the survey. The survey consisted of 58 questions grouped into pre-operative, acute care and post-acute rehabilitation. Variation of practice was described in terms of number, duration and type of visits along with goals of care and program delivery methods.

**Results:**

Of the 80 respondents, 26 (33 %) stated they worked in small centres or rural settings in Alberta with the remaining respondents working in two large urban sites. The primary treatment goal differed for each phase across the continuum of care in that pre-operative phase was directed at improving muscle strength, functional activities were commonly reported for acute care, and post-acute phase was directed at improving joint range-of-motion. Proportionally, more physiotherapists from rural areas treated patients in out-patient hospital departments (59 %), whereas a higher proportion in urban physiotherapists saw patients in private clinics (48 %). Across the continuum of care, treatment was primarily delivered on an individual basis rather than in a group format.

**Conclusions:**

Variation of practice reported with pre-and post-operative care in the community will stimulate dialogue within the profession as to what is the minimal standard of care to provide patients undergoing TJA.

**Electronic supplementary material:**

The online version of this article (doi:10.1186/s12913-016-1873-9) contains supplementary material, which is available to authorized users.

## Background

Physiotherapy is an essential service in preparing for and recovering after total hip (THA) and knee arthroplasty (TKA) because it has been shown to improve pain, function and health-related quality of life (HRQL) [1, 2]. Current evidence also emphasizes the need for rehabilitation to optimize patient outcomes before and after surgery [3, 4], reduce healthcare costs [5] and increase patient satisfaction [6]. Inadequate rehabilitation is likely a major contributor to residual pain [7], muscle weakness, functional limitations and poor HRQL reported in approximately 25 % of individuals after total joint arthroplasty (TJA) [8–10]. Comprehensive and timely rehabilitation for TJA is needed to maximize recovery from this elective surgical procedure for hip and knee arthritis [11].

During 2012–2013, 41,910 primary THA and 52,868 primary TKA procedures were performed in Canada [12], of which the Alberta provincial rate for THA was 163.7.2 per 100,000 and 202.3 per 100,000 for TKA in 2012–2013 [12]. With the aging population, increased prevalence of osteoarthritis (OA) and growing demand for TJA procedures [13, 14], related rehabilitation services will have an increasingly greater impact on health care systems. Despite evidence supporting TJA rehabilitation for OA and the presence of best practice recommendations [1, 2, 15–18], marked variations in current practice exists [19–21]. Practice variation in other areas of health care, such as back surgery, has been associated with excessive healthcare costs and outcome variability [22].

One approach to standardize care and provide evidence-based care is the implementation of clinical pathways. Within Alberta, provincial multidisciplinary clinical pathways for THA and TKA have been in existence for acute care management over the past decade. The clinical pathways include medical, pharmaceutical and rehabilitation management directed at a 3 to 4 day length of stay (LOS) in acute care settings. The primary aim of implementing clinical pathways for THA and TKA in Alberta was to standardize care and provide best practice [23]. In spite of guidelines for rehabilitation of TJA, no clinical pathways exist for pre-operative or post-acute care within the province. With more recent healthcare reform, little is known about the practice variation of physiotherapy for THA and TKA. Although the overall success of recovery from surgery for TJA is dependent upon many factors including rehabilitation, the optimal type and frequency of physiotherapy has not been established.

The healthcare landscape is changing and physiotherapy services are not always appropriately prioritized relative to other services [24]. Physiotherapists typically provide treatment for TJA in acute care hospitals, rehabilitation facilities, and within the community (eg. out-patient hospital, home care and private practice) which makes evaluation of rehabilitation services across the continuum of care challenging. Because administrative data do not capture the variation of treatment visit variation (eg. number, duration, type) for rehabilitation services across the continuum of care, a survey of physiotherapists may provide better insight into the services provided. The primary objective of this cross-sectional survey was to describe the reported practice of physiotherapy for TJA across the continuum of care within the context of a provincial TJA clinical pathway and highlight possible gaps in care.

## Methods

This cross-sectional on-line survey was accessible to licensed physiotherapists in Alberta, Canada for 11 weeks from June 6, 2014 – September 22, 2014. The invitation to participate was posted on the provincial professional college and association web-site (http://www.physiotherapyalberta.ca/). All practicing physiotherapists in the province are licensed by the provincial professional college and association. Physiotherapists who treated at least five patients with TJA annually regardless of practice setting, were asked to complete the survey. Variation of practice was described in terms of number, duration and type of visits along with goals of care and program delivery methods.

In 2013, 2345 physiotherapists were licensed to practice in the province (2013 pop 4,007,700) [25]. Approximately 80 % (*n* = 1873) of physiotherapists were from the two largest metropolitan areas in the provinces, Calgary and Edmonton [26]. An estimated 1580 (66 %) of licensed therapists reported practice areas (general, rheumatology, orthopaedics) that may have included treating patients with TJA. Approximately 47 % (*n* = 1035) of licensed physiotherapists were in private practice and another 29 % (*n* = 645) practiced in general hospitals [26]. Twelve centres in Alberta perform surgery for TJA; six high volume hospitals in large metropolitan areas (Edmonton, Calgary) and six smaller centres (Camrose, Weslock, Grande Prairie, Red Deer, Medicine Hat and Lethbridge) [27]. Approximately 70 % of TJA surgeries were performed in the large metropolitan areas (Calgary and Edmonton).

Within the acute care setting, the provincial pathway was developed for a 3 to 4 day length of stay for both THA and TKA and included physiotherapy services. The rehabilitation component of the pathway included commencement of mobilization of patients on the day of surgery. Mobilization can range from sitting at the edge of the bed, to standing or walking. Within the clinical pathway, mobility was recommended twice daily. Although the clinical pathway outlines physiotherapy activities during acute-care, no pre-established standardized guidelines exist for pre-operative and post-discharge rehabilitation services. Service delivery is dependent upon available funding within each of the five provincial health zones. Seven pre-operative and seven post-acute visits for TJA are covered by the regional health zones in Calgary and Edmonton health zones. Within the other three health zones (North, Central, South), rehabilitation services are dependent upon available human resources in the community. In the North and Central Zones, seven visits are covered by the health zone for post-acute care if private practice physiotherapy exists in the community. Physiotherapy in the South Zone does not provide coverage for private practice services for TJA but rather patients receive their rehabilitation at out-patient hospital sites. The number of out-patient hospital physiotherapy visits is dependent upon the therapist’s discretion.

The online survey consisted of 58 questions related to care in the pre-operative (21 questions), acute care (20 questions) and post-acute (17 questions) phases of care. (Additional file 1) The survey questions addressed TKA and THA individually and questions were categorized with respect to the three phases of the continuum of care: pre-operative, acute care and post-acute rehabilitation. Survey development was based on earlier unpublished work done by one of the authors (MDW) in British Columbia (BC) and expert opinion in 2006. The unpublished work was based on paper-based questionnaires which were distributed through e-mail to rehabilitation practice leaders across the five provincial health authorities within BC. Questionnaire topics included information on any pre-operative, acute care or outpatient (post-acute) rehabilitation services provided including patient volume, treatment format and time, specific interventions, discharge criteria and follow-up care. Thirty clinical BC sites completed the questionnaires.

The questions that dealt with each phase of care covered a broad range of topics including: goals of care, program delivery methods, the structure and timing of the program and the physiotherapist’s perception of the program’s effectiveness. Most questions were close-ended (multiple-choice, ranking, or “indicate all that apply” options) to standardize responses and improve survey completion [28]. The survey was created using the Survey Monkey tool [29]. Ethics approval was obtained from the Health Research Ethics Review Board of the University of Alberta. Because this was an anonymous cross-sectional survey, participants provided implicit consent by completing the on-line survey.

### Analysis

Summary statistics (e.g., frequencies, proportions, means, standard deviations) were completed for responses with stratification of urban and rural settings. Statistical analyses were performed using SPSS version 22.0.

## Results

Overall, 83 participants responded based on the requirement that they have treated at least 5 patients with TJA over the past year. Three entries were omitted because less than three questions were answered for each case. Of the remaining 80 respondents, 39 stated they provided preoperative care, 30 operative care and 45 post-acute care. Of this sample, 45 (46 %) respondents provided care in one of the three practice settings and 5 (6 %) respondents provided care across the continuum of care (pre-operative, acute and post acute care). There were 26 (33 %) respondents who worked in small centres or rural settings in Alberta with the remaining respondents working in the two large urban sites.

### Pre-operative care

Of the 80 respondents, 39 (49 %) reported providing pre-operative care to patients. The majority of physiotherapists who provided pre-operative care, 30 (79 %) delivered both exercise and education in the session while eight (21 %) provided education alone. These sessions were typically provided on an individual basis (85 %) within 3 months of scheduled surgery and occurred either in the hospital or clinic. Half of respondents reported providing only one session for the pre-operative program while 30 % provided two to four pre-operative sessions. Only six (20 %) stated they evaluated patient satisfaction of the program.

Of the 39 respondents who stated they treated patients during the pre-operative phase, 27 respondents ranked four general treatment goals from a pre-generated list for a pre-operative program. (Supplemental material) Almost half ranked the primary treatment goal to improve strength (13, 48 %), whereas pain control was ranked as the primary treatment goal by 4 respondents (Table 1). When asked about the type of exercises offered, resistive training using free weights or resistive bands were more often reported (26, 96 %), although functional activities (23, 85 %), and balance activities (20, 74 %) were also performed (Table 2). Respondents reported that treatment was typically based on past clinical experience or clinical guidelines.

**Table 1:** Distribution of respondents ranking primary treatment goal by phase of care

	Pre-operative (*n* = 27)	Acute care (*n* = 30)	Post-acute (*n* = 45)
Improve range of motion	4 (18.5 %)	3 (10 %)	19 (42 %)
Improve muscle strength	13 (48 %)	1 (3 %)	1 (2 %)
Improve function	5 (15 %)	17 (57 %)	16 (36 %)
Pain control	5 (18.5)	9 (30 %)	9 (20 %)

**Table 2:** Treatment offered by phase of care in the community

Type of Treatment	Pre-operative (*n* = 27)	Post-acute (*n* = 45)
Strengthening	26 (96 %)	41 (91 %)
Functional activities	23 (85 %)	43 (96 %)
Balance retraining	20 (74 %)	39 (87 %)
Cardiovascular retraining	13 (48 %)	23 (51 %)
Flexibility/stretching	3 (11 %)	8 (18 %)

The three most commonly reported types of education for these pre-operative sessions were education that focussed on exercise (*n* = 30), movement precautions (*n* = 25) and pain control (*n* = 25). Exercise handouts and education booklets were frequently provided. The majority of in-person education sessions were directed by a health professional (*n* = 29), although a few were presented in the form of a DVD (*n* = 6).

### Acute care

Of the 30 (37 %) respondents who stated that they provided acute care treatment for patients after their surgery, 18 (60 %) were from urban centres. The majority of respondents from rural settings typically had one physiotherapist, one or two physiotherapy assistants and one occupational therapist working on the unit. The units in the rural settings were smaller than the urban centres ranging from six to 45 beds, whereas the urban respondents stated they were working on units with up to 120 beds. The reported average LOS for both THA and TKA was 4 days. All respondents stated that they followed the provincial clinical pathway with all physiotherapy visits starting on the day of surgery or post-op day one. One or two visits per day by the physiotherapist occurred whereas the physiotherapy assistants visited two or three times per day for the duration of the hospital stay.

When asked to rank treatment goals in order of importance, 57 % of respondents ranked performing functional activities as the primary goal in acute care, followed by pain control (30 %) (Table 1). The first session of therapy included dangling at the edge of bed and walking. All respondents stated that patients were encouraged to walk daily. Patients practised a variety of exercises during the in-patient stay ranging from bed, sitting, standing and walking activities. Ice and elevation were the primary modalities used when dealing with swelling. Six respondents stated they used continuous passive motion after TKA.

### Post-acute care

Of the 45 respondents who provided post-acute care, the majority were from private practice (38 %), followed by out-patient hospital departments (33 %), subacute facilities or provincial hip and knee clinics (18 %), and home care (11 %). Proportionally, more physiotherapists from rural areas treated patients in out-patient hospital departments (59 %), whereas a higher proportion in urban physiotherapists saw patients in private clinics (48 %). Rural therapists reported seeing patients at a mean 2.9 (SD 1.6) weeks post-operatively for TKA and at a mean 3.9 (SD 2.1) weeks for THA. These mean times were in comparison to the urban therapists who reported seeing patients with TKA at a mean 3.7 (SD 2.3) weeks post-operatively and mean 4.9 (SD 2.1) weeks for THA (Table 3). Rural therapists typically saw patients for fewer sessions but reported longer follow-up sessions with patients.

**Table 3:** Reported post-operative physiotherapy sessions by rural and urban settings

	Total Hip Arthroplasty		Total Knee Arthroplasty	
	Urban mean (95 % CI)	Rural mean (95 % CI)	Urban mean (95 % CI)	Rural mean (95 % CI)
Number of sessions	8.5 (6.5,10.5)	6.1 (4.3,7.9)	9.4 (7.0,11.8)	7.2 (5.4,9.0)
Time from hospital discharge to start of post-operative physiotherapy (wks)	4.9 (3.8,5.9)	3.9 (2.6,5.1)	3.7 (2.6,4.9)	2.9 (2.0,3.8)
Duration of initial assessment (min)	44.2 (33.5,54.9)	45.0 (37.2,52.8)	44.1 (33.5,54.9)	45.0 (37.2,52.8)
Duration of follow-up sessions (min)	28.6 (20.8,36.5)	35.3 (26.9,43.8)	28.6 (20.8,36.5)	37.2 (28.2,46.4)

During post-acute care, regardless of urban or rural settings, 42 % of physiotherapists rated improving ROM as the primary treatment goal for TJA, 36 % rated improving function as the primary goal, and 20 % of respondents rated pain control as a primary treatment goal (Table 1). The most common types of exercises reported included functional training (96 %) and strengthening exercises with free weights, body weight and/or strengthening machines (91 %). Cardiovascular training was the least commonly reported type of exercise (51 %) (Table 2). Resources to guide exercises were primarily based on established guidelines (91 %) and past clinical experience (89 %). Commonly used modalities for TJA included ice (61 %), interferential current (39 %), heat (37 %), and muscle stimulation (33 %). The majority of treatment (91 %) was provided on an individual level with 2–3 rural respondents reported conducting small group classes.

### Continuum of care

The primary treatment goal differed for each phase across the continuum of care. The pre-operative phase was directed at improving muscle strength, whereas functional activities were commonly mentioned for acute care and post-acute phase was directed at improving joint ROM. Treatment also differed across the continuum of care in that strengthening exercises such as the use of free weights were most frequently practiced during the pre-operative phase; bed-sitting-standing activities were the most commonly reported activities during the acute phase and functional strengthening activities such as stair climbing were most frequently reported during the post-acute phase. Across the continuum of care, treatment was primarily delivered on an individual basis rather than in a group format.

## Discussion

Findings from this cross-sectional survey suggest that variation of rehabilitation across the continuum of care and geographic locations exists for TJA within the jurisdiction of provincial healthcare system. Variation in practice was reported with the treatment goals and type of treatment reported in each phase of care. It was expected that variability would occur across the three phases of care, pre-operative, acute and post-operative care, because patients’ needs differ with each phase. What was not known prior to this study was the variability in treatment visit timing, duration, and type of physiotherapy services delivered at a province level. A better understanding of treatment variability is essential so that gaps in care can be identified and transparency in health care promoted. Variation of treatment was more pronounced with pre and post-operative care than with acute care where greater consensus was reported by the physiotherapists. This was likely due to the clinical acute care pathway which has been established provincially for a number of years. The clinical pathway is a means of standardizing care and managing LOS. It also is a tool to establish expectations of care for both the patient/family and the healthcare members [5]. While the clinical pathway has been designed to ensure standardized care within acute care, standardized care within in the community has not been developed within the health regions or province.

The frequency and type of services provided by physiotherapists varied in the community both before and after surgery. Despite the evidence supporting TJA rehabilitation for improved patient outcomes and the availability of best practice recommendations [30], marked variations in clinical practice have also been documented by others [19–21, 31]. Because of documented variation in practice, others have advocated that quality assessment of health care, including rehabilitation, should be systematically assessed and evaluated [32]. It should be acknowledged, however, that practice variation at the therapist level is affected by macro level factors including the healthcare structure, financial reimbursement schemes, human resources and cultural influences [19]. Clinical pathways instituted at the provincial level were a means of standardizing care. This may explain, in part, as to why greater consensus in treatment goals and offered treatment was reported by physiotherapists for acute care management. While the clinical pathways for TJA in Alberta do not extend beyond the acute care setting, quality indicators may be one means to reduce practice variation in the community. Quality indicators go beyond clinical pathways to promote quality care and provide a means to measure and benchmark whether minimum standards of care have been met. To date no quality indicators exist for rehabilitation of TJA; however, work is currently underway to develop quality indicators for TJA [33].

Variation of the frequency and duration of sessions was reported between urban and rural practice settings which could be affected by financial and human resources [19]. An indication of limited capacity in rural regions that was seen in this survey was a trend toward fewer therapy sessions reported by rural physiotherapists relative to the physiotherapists in urban settings. The pressure on providing physiotherapy in the community was also felt with the decreasing LOS for TJA, which has shifted the provision of rehabilitation services from the acute care setting into the community where little standardization in care exists [23, 34]. While the focus of the acute care pathway is to prepare patients for discharge, little focus has been directed toward the delivery of services in the community once the patient has been discharged. No clinical pathway or quality indicators exist in the community for rehabilitation services [23]. Future work is required to determine how post discharge services should be organized and identify approaches to optimize patient outcomes. Quality indicators which provide a benchmark of whether minimum standards of care are being met, may be a feasible approach to determine rehabilitation services for TJA in the community.

While we did not specifically examine the financial and human resources within this survey, it is likely that reimbursement was linked with services rendered and the available human resources. Others have reported that the ability or willingness to pay as barriers to access to private community physiotherapy services in Alberta [35]. Coverage for rehabilitation services in the community varies by health zones and, at best, consists of a finite number of visits that do not cover the duration of rehabilitation. Thus, individuals must pay for service or have access to third party coverage for further treatment. With capped visits for community rehabilitation, this negates the rehabilitation needed to optimize patient recovery within the first 3 to 6 months. As the demand for TJA continues to increase with an aging population and increasing prevalence of OA, health system efficiency and resource allocation is needed for rehabilitation of TJA.

The strength of this study lies in the broad spectrum of responses we received from physiotherapists who practiced in both urban and rural settings in all practice sectors across the continuum of care. Although this self-report information may be prone to reporting bias, it sheds light on the type of care, goals and treatment for TJA. Overall limitations of this study primarily pertain to the cross-sectional design of the survey. Response was voluntary and was limited to the number of licensed physiotherapists in the province. Notwithstanding, there is no established mechanism within the provincial data to identify the number of physiotherapists who treat patients with TJA and the type of services they provide. The intent of this survey was to provide preliminary information regarding the type of services delivered by physiotherapists for patients undergoing TJA across the continuum of care. The reported variation of practice seen pre-and post-operatively in the community will stimulate dialogue within healthcare as to what is the minimal standard of rehabilitation to provide patients recovering from TJA.

## Conclusions

Findings from this survey provide insight into the rehabilitation care that patients receive for TJA across the continuum of care. Standardized care was reported by physiotherapists in acute care even in different sized settings with different levels of service. More investigation, however, is needed to determine the optimal preoperative and post acute patient-centred care for rehabilitation delivered in urban and rural practice settings.

## Additional file

Additional file 1: On-line Survey. (PDF 1971 kb)

## Abbreviations

HRQL: Health-related quality of life
LOS: Length of stay
OA: Osteoarthritis
THA: Total hip arthroplasty
TJA: Total joint arthroplasty
TKA: Total knee arthroplasty

## References

[1] Minns Lowe CJ, Barker KL, Dewey M, Sackley CM (2007). Effectiveness of physiotherapy exercise after knee arthroplasty for osteoarthritis: systematic review and meta-analysis of randomised controlled trials. BMJ.

[2] Coulter CL, Scarvell JM, Neeman TM, Smith PN (2013). Physiotherapist-directed rehabilitation exercises in the outpatient or home setting improve strength, gait speed and cadence after elective total hip replacement: a systematic review. J Physiother.

[3] Westby MD (2012). Rehabilitation and total joint arthroplasty. Clin Geriatr Med.

[4] Wallis JA, Taylor NF (2011). Pre-operative interventions (non-surgical and non-pharmacological) for patients with hip or knee osteoarthritis awaiting joint replacement surgery--a systematic review and meta-analysis. Osteoarthritis Cartilage.

[5] Fancott C, Jaglal S, Quan V, Berg K, Cott CA, Davis A (2010). Rehabilitation services following total joint replacement: a qualitative analysis of key processes and structures to decrease length of stay and increase surgical volumes in Ontario, Canada. J Eval Clin Pract.

[6] Westby MD, Backman CL (2010). Patient and health professional views on rehabilitation practices and outcomes following total hip and knee arthroplasty for osteoarthritis:a focus group study. BMC Health Serv Res.

[7] Beswick AD, Wylde V, Gooberman-Hill R, Blom A, Dieppe P (2012). What proportion of patients report long-term pain after total hip or knee replacement for osteoarthritis? A systematic review of prospective studies in unselected patients. BMJ Open.

[8] Meier W, Mizner RL, Marcus RL, Dibble LE, Peters C, Lastayo PC (2008). Total knee arthroplasty: muscle impairments, functional limitations, and recommended rehabilitation approaches. J Orthop Sports Phys Ther.

[9] Bhave A, Mont M, Tennis S, Nickey M, Starr R, Etienne G (2005). Functional problems and treatment solutions after total hip and knee joint arthroplasty. J Bone Joint Surg Am.

[10] Trudelle-Jackson E, Emerson R, Smith S (2002). Outcomes of total hip arthroplasty: a study of patients one year postsurgery. J Orthop Sports Phys Ther.

[11] Bumpass DB, Nunley RM (2012). Assessing the value of a total joint replacement. Curr Rev Musculoskelet Med.

[12] Canadian Institute for Health Information. Hip and Knee Replacements in Canada: Canadian Joint Replacement Registry 2014 Annual Report. Ottawa: Canadian Institute for Health Information; 2015. https://secure.cihi.ca/free_products/CJRR 2014 Annual Report_EN-web.pdf.

[13] Tian W, DeJong G, Brown M, Hsieh CH, Zamfirov ZP, Horn SD (2009). Looking upstream: factors shaping the demand for postacute joint replacement rehabilitation. Arch Phys Med Rehabil.

[14] Antoniou J, Martineau PA, Filion KB, Haider S, Zukor DJ, Huk OL (2004). In-hospital cost of total hip arthroplasty in Canada and the United States. J Bone Joint Surg Am.

[15] Fernandes L, Hagen KB, Bijlsma JW, Andreassen O, Christensen P, Conaghan PG (2013). EULAR recommendations for the non-pharmacological core management of hip and knee osteoarthritis. Ann Rheum Dis.

[16] Hochberg MC, Altman RD, April KT, Benkhalti M, Guyatt G, McGowan J (2012). American College of Rheumatology 2012 recommendations for the use of nonpharmacologic and pharmacologic therapies in osteoarthritis of the hand, hip, and knee. Arthritis Care Res.

[17] Jordan KM, Arden NK, Doherty M, Bannwarth B, Bijlsma JW, Dieppe P (2003). EULAR Recommendations 2003: an evidence based approach to the management of knee osteoarthritis: Report of a Task Force of the Standing Committee for International Clinical Studies Including Therapeutic Trials (ESCISIT). Ann Rheum Dis.

[18] Pendleton A, Arden N, Dougados M, Doherty M, Bannwarth B, Bijlsma JW (2000). EULAR recommendations for the management of knee osteoarthritis: report of a task force of the Standing Committee for International Clinical Studies Including Therapeutic Trials (ESCISIT). Ann Rheum Dis.

[19] Lingard EA, Berven S, Katz JN, Kinemax Outcomes G (2000). Management and care of patients undergoing total knee arthroplasty: variations across different health care settings. Arthritis Care Res.

[20] Mahomed NN, Lau JT, Lin MK, Zdero R, Davey JR (2004). Significant variation exists in home care services following total joint arthroplasty. J Rheumatol.

[21] Freburger JK, Holmes GM, Ku LJ, Cutchin MP, Heatwole-Shank K, Edwards LJ (2011). Disparities in post-acute rehabilitation care for joint replacement. Arthritis Care Res.

[22] Mulley AG (2009). Inconvenient truths about supplier induced demand and unwarranted variation in medical practice. BMJ.

[23] Gooch K, Marshall DA, Faris PD, Khong H, Wasylak T, Pearce T (2012). Comparative effectiveness of alternative clinical pathways for primary hip and knee joint replacement patients: a pragmatic randomized, controlled trial. Osteoarthritis Cartilage.

[24] Landry MD, Goldstein M, Stokes E (2012). Physiotherapy health services research (PHSR): the road ‘that must now be taken’. Physiother Res Int.

[25] Statistics Canada. CANSIM table: Population by year, by province and territory. 2015. http://www.statcan.gc.ca/tables-tableaux/sum-som/l01/cst01/demo02a-eng.htm.

[26] College of Physical Therapists of Alberta. Annual Report 2013. 2014. http://www.physiotherapyalberta.ca/files/annual_report_2013.pdf.

[27] Alberta Health Services Zones. 2-17-2015. Online Source. http://www.albertahealthservices.ca/ahs-map-ahs-zones.pdf.

[28] Kitchneham BA, Pfleeger SL. Principles of survey research Part 3: Constructing a survey instrument. 27, 20–24. 2002. ACM Sigsoft. Software Engineering Notes.

[29] Survey Monkey. Online Source. 2014. https://www.surveymonkey.com/. Accessed 2016.

[30] Westby MD, Brittain A, Backman CL (2014). Expert consensus on best practices for post-acute rehabilitation after total hip and knee arthroplasty: a Canada and United States Delphi study. Arthritis Care Res.

[31] Roos EM (2003). Effectiveness and practice variation of rehabilitation after joint replacement. Curr Opin Rheumatol.

[32] Gronhaug G, Osteras N, Hagen KB (2014). Quality of hip and knee osteoarthritis management in primary health care in a Norwegian county: a cross-sectional survey. BMC Health Serv Res.

[33] Westby M, Klemm A, Li L, Jones CA. The emerging role of quality indicators in physical therapy practice and health services delivery. Phys Ther. 2016; 96:90-100. doi:10.2522/ptj.20150106. 10.2522/ptj.20150106 PMC4706598 26089040

[34] Cott CA, Davis AM, Badley EM, Wong R, Canizares M, Li LC (2016). Commonalities and differences in the implementation of models of care for arthritis: key informant interviews from Canada. BMC Health Serv Res.

[35] Davis AM, Cott C, Landry MD, Li L, Jones A, Linneker S et al. Care for people with arthritis. Policy: decisions, impacts and gaps. Edited by Arthritis Community and Evaluation Unit. MOCA2010-07/005. 2010. Toronto, ON. http://www.modelsofcare.ca/pdf/10-05.pdf.

