# Supplementary material for: Total joint arthroplasty: practice variation of physiotherapy across the continuum of care in Alberta
Source: BMC Health Serv Res. 2016 Nov 4;16:627. doi: 10.1186/s12913-016-1873-9 (PMC5095989; doi:10.1186/s12913-016-1873-9)
Supplement: Additional file 1: — On-line Survey. (PDF 1971 kb) [file 12913_2016_1873_MOESM1_ESM.pdf]

1. In what region of the province do you primarily work?

Pre-operative Program

2. Do you provide a pre-operative rehabilitation and/or education program for total hip/knee clients?

- ◯ Yes
- ◯ No

3. What do you offer in you pre-operative program?

- ◯ Education and Exercise
- ◯ Education only

4. What are the goals of your PRE-OPERATIVE program? Rank your responses with 1 being the highest priority and 4 being the lowest priority

☐	Improving R.O.M	☐ N/A
☐	Improving strength	☐ N/A
☐	Improving function	☐ N/A
☐	Pain control	☐ N/A

5. What forms of exercise do you offer your clients? (Check all that apply)

- ☐ UE/LE strengthening using free weights/bands/bodyweight/or machines
- ☐ Functional training (i.e. stair walking, sit-stand)
- ☐ Core exercises
- ☐ Balance/weight transfer training
- ☐ Cardio exercise
- ☐ Other (please specify)

6. What resources do you primarily use to guide your exercise intervention? (Check all that apply)

- ☐ Past clinical experience
- ☐ Established practice guidelines
- ☐ Online exercise databases
- ☐ Other (please specify)

7. When planning exercise for clients, preference is given to:

- ◯ Open kinetic chain exercises
- ◯ Closed kinetic chain exercises
- ◯ Both

8. In what setting does the exercise take place?

- ◯ A group setting
- ◯ An individualized session
- ◯ Both

9. How far in advance of surgery is pre-operative exercise provided to your clients?

Pre-Operative Education

10. Please indicate the setting in which you provide pre-op rehabilitation/education:

- [ ] Hospital
- [ ] Client's home
- [ ] Community centre
- [ ] Outpatient clinic
- [ ] Other (please specify)

11. Which of the following professionals participate in the pre-op rehab program? (Check all that apply)

- [ ] Occupational Therapist
- [ ] Physical Therapist
- [ ] Therapy Assistant
- [ ] Social Worker
- [ ] Community Case Manager
- [ ] Nurse
- [ ] Physician/Surgeon
- [ ] Other (please specify)

12. During the pre-operative program, on which of the following do you provide education? (Check all that apply)

- [ ] Exercise
- [ ] Movement Precautions
- [ ] Meals and household management
- [ ] Equipment recommendations
- [ ] Transportation
- [ ] Car Transfers
- [ ] Performing transfers
- [ ] Emotional impact of undergoing procedure
- [ ] Ability to manage self-care
- [ ] Pain control
- [ ] Post-op medical protocols (i.e. administration of blood thinners)
- [ ] Average length of hospital stay
- [ ] Caregiver role
- [ ] Physical home environment (i.e. potential need to modify)
- [ ] Other (please specify)

13. Which of the following educational resources do you provide your clients with pre-operatively: (Check all that apply)

- [ ] Education booklet
- [ ] Exercise handouts
- [ ] Community service(s) information sheet
- [ ] Equipment list
- [ ] List of equipment loan cupboards and/or local medical equipment vendors
- [ ] Service provider phone number list
- [ ] Online resources
- [ ] Other (please specify)

14. Does pre-operative education occur in:

- ◯ A group setting
- ◯ An individualized session
- ◯ Both

15. In what format is the session presented?

- ☐ In person by health professionals
- ☐ In person by non-health professionals
- ☐ DVD format with questions afterward
- ☐ Other (please specify)

16. How many people typically present to the group or individual?

- ◯ 1 presenter
- ◯ 2 presenters
- ◯ More than 2 presenters
- ◯ Other (please specify)

17. How many sessions does the individual attend?

- ◯ 1
- ◯ 2 - 4
- ◯ 5 or more
- ◯ Other (please specify)

18. Please identify which of the following elements your clients have an opportunity to practice, with a therapist, prior to their admission to hospital. (Check all that apply)

- ☐ Exercise
- ☐ Adhering to movement precautions
- ☐ Proper use of gait aids
- ☐ Performing transfers
- ☐ Strategies to manage pain
- ☐ Other (please specify)

19. Have you recently evaluated your client's satisfaction with your pre-op education program?

- ◯ Yes
- ◯ No

20. If "yes" when did you evaluate their satisfaction?

- ◯ After the pre-operative program
- ◯ After the surgery
- ◯ Both

21. Did you change any elements of your program as a result of client feedback?

22. How far in advance of surgery is pre-operative education provided to your clients?

Inpatient Acute Phase

23. Do you provide acute in-patient care for patients with total hip/knee replacement surgeries?

- ◯ Yes
- ◯ No

24. What are your goals for the IN-PATIENT acute phase? Rank your responses with 1 being the highest priority and 4 being the lowest priority

Rank	Goal	N/A
☐	Improving R.O.M	☐ N/A
☐	Improving strength	☐ N/A
☐	Improving function	☐ N/A
☐	Pain control	☐ N/A

25. Does your facility follow a care map or clinical pathway for all total hip/knee replacement surgeries?

- ◯ Yes
- ◯ No

26. What is the average length of stay in DAYS for patients in acute care:

Total Knee clients ________

Total Hip clients ________

27. Indicate the number of times per day, on average, clients are seen by each of the following team members:

	Visits per Day
Physical Therapist	☐
Occupational Therapist	☐
Therapy Assistant	☐
Recreation Therapist/ Health Care Aid	☐

28. Which are the following exercises are performed during in-patient stay? (Check all that apply)

- ☐ Bed exercises
- ☐ Seated exercises
- ☐ Standing exercises

29. How frequently are supervised exercises performed each day?

- ◯ 1
- ◯ 2
- ◯ 3
- ◯ 4+

30. How often are patients instructed to perform exercises independently each day?

- ◯ 1
- ◯ 2
- ◯ 3
- ◯ 4
- ◯ 5+
- ◯ Other (please specify)

____

31. How many days after surgery are exercises initiated following total hip or knee replacement?

- ◯ 0
- ◯ 1
- ◯ 2
- ◯ 3
- ◯ 4

32. Is each patient required to walk daily?

- ◯ Yes
- ◯ No

33. If "Yes":

How many times each day are clients assisted with walking?	
On which day post-op is dangling initiated?	
On which day post-op is walking initiated?	

Other (please specify)

34. Please indicate which of the following functions form the PRIMARY roles for each of the following team members:

	Physical Therapy	Therapy Assistant
Discharge planning	☐	☐
Mobility (i.e. walking)	☐	☐
Bed exercises	☐	☐
Transfers: Lie-Sit and Sit-Stand	☐	☐
Caregiver education	☐	☐
Gathering info re: home environment	☐	☐
Referral to in-and out-patient rehab	☐	☐
Education on care of the incision	☐	☐

Other (please specify)

35. Following a normal care path, on which day post-operative are the following functions initiated post total hip or knee replacement?

	Day
Discharge planning	
Mobility (i.e. walking)	
Bed exercises	
Seated exercises	
Standing exercises	
Transfers: Lie-Sit and Sit-Stand	
Car transfers	
Caregiver education	
Gathering info re: home environment	
Referral to in-and out-patient rehab	
Education on care of the incision	

36. Please indicate which of the following modalities are used during the client's in-patient stay to manage pain and swelling: (Check all that apply)

- [ ] Ice therapy
- [ ] Heat therapy
- [ ] Elevation
- [ ] Sequential Pressure cuff
- [ ] TENS
- [ ] Pain medications
- [ ] CPM
- [ ] Other (please specify)

37. Please indicate which of the following modalities are used to increase strength (Check all that apply):

- [ ] Biofeedback
- [ ] Bed/seated/standing exercises
- [ ] Functional exercise (i.e. practicing transfers, functional activities, LE exercises)
- [ ] Muscle stimulation
- [ ] Other (please specify)

38. Please identify the exercises typically used with patients: (Check all that apply)

- [ ] Bed mobility exercises
- [ ] Bed exercises
- [ ] Seated exercises
- [ ] Standing exercises
- [ ] Gait exercises
- [ ] Stair walking
- [ ] ROM exercises
- [ ] Other (please specify)

39. Please indicate which team members perform the following elements of discharge planning:

	Physical Therapist	Occupational Therapist	Community Case Manager	Nurse	Social Worker
Arranging equipment for client's home	☐	☐	☐	☐	☐
Practicing stairs	☐	☐	☐	☐	☐
Evaluating ADL performance	☐	☐	☐	☐	☐
Addressing meals and household management	☐	☐	☐	☐	☐
Making referral to community care (if needed)	☐	☐	☐	☐	☐
Referrals to in-patient/out-patient rehabilitation programs	☐	☐	☐	☐	☐
Ensuring client has transportation home	☐	☐	☐	☐	☐

Other (please specify)

***40. Upon discharge, where are the total hip clients routinely referred to for post-discharge follow-up? Please rank the following from 1-3, with 1 being the most frequent referral made upon discharge and 3 indicating the least frequent discharge referral.**

☐	Out-patient Rehabilitation (hospital based)	☐ N/A
☐	Privately run out-patient clinic	☐ N/A
☐	In-patient Rehabilitation Unit subacute	☐ N/A

***41. Upon discharge, where are the total knee clients clients routinely referred to for post-discharge follow-up? Please rank the following from 1-4, with 1 being the most frequent referral made upon discharge and 4 indicating the least frequent discharge referral.**

☐	Community Therapy	☐ N/A
☐	Out-patient Rehabilitation (hospital based)	☐ N/A
☐	Privately run out-patient clinic	☐ N/A
☐	In-patient Rehabilitation Unit subacute	☐ N/A

42. Please provide the following information:

Number of Occupational Therapists (in FTE's) working on in-patient orthopedic unit	
Number of Physical Therapists (in FTE's) working on in-patient orthopedic unit	
Number of Rehab Assistants (in FTE's) working on in-patient orthopedic unit	
Average number of total joint clients admitted to unit on a MONTHLY basis	
Total number of beds on in-patient orthopedic unit	

Outpatient Rehabilitation

43. Do you offer out-patient rehabilitation?

- ○ Yes
- ○ No

***44. What are the goals of your POST-OPERATIVE program? Rank your responses with 1 being the highest priority and 4 being the lowest priority.**

☐	Improving R.O.M	☐ N/A
☐	Improving strength	☐ N/A
☐	Improving function	☐ N/A
☐	Pain control	☐ N/A

45. In which out-patient rehabilitation setting do you provide your services?

- ○ Private Clinic
- ○ Hospital Based Setting
- ○ Home care
- ○ Other (please specify)

46. Please indicate which of the following modalities are used in treating total joint replacement patients in the out-patient clinic: (Check all that apply)

- ☐ Ice (bagged from machine)
- ☐ Cryocuff
- ☐ Ice packs
- ☐ Hot packs
- ☐ Muscle stimulation
- ☐ TENS
- ☐ Interferential Current
- ☐ Biofeedback
- ☐ Other (please specify)

47. What forms of exercise do you offer your patients? (Check all that apply)

- [ ] Strengthening exercises using free weights/bands/bodyweight/or machines
- [ ] Functional training (i.e. stair walking, sit-stand)
- [ ] Core exercises
- [ ] Balance/weight transfer training
- [ ] Cardio exercise

Other (please specify)

48. What resources do you primarily use to guide your exercise intervention? (Check all that apply)

- [ ] Past clinical experience
- [ ] Established practice guidelines
- [ ] Online exercise databases
- [ ] Other (please specify)

49. When planning exercise for patients, preference is given to:

- ○ Open kinetic chain exercises
- ○ Closed kinetic chain exercises
- ○ Both

50. Are patients seen in a group setting?

	Yes	No	N/A
Total Hip clients	○	○	○
Total Knee clients	○	○	○

51. If you answered "yes", how many patients are seen per group?

Total Hip group: number of clients per group:

Total Knee group: number of clients per group:

52. Who is the PRIMARY person responsible for the bookings for appointments at your clinic? (Check all that apply)

- ○ Clerk/Admin Assistant
- ○ Rehabilitation Assistant
- ○ Physical Therapist or Occupational Therapist
- ○ Other (please specify)

53. If patients are seen for individual treatment sessions, what is the average number of treatment sessions provided?

Total Hip clients: Avg number of sessions:

Total Knee clients: Avg number of sessions:

54. How much time is scheduled for the following activities in your clinic?

Total Hip clients: Initial assessment (in minutes per session)

Total Knee clients: Initial assessment (in minutes per session)

Total Hip clients: Follow-up visit (in minutes per session)

Total knee clients: Follow-up visit (in minutes per session)

55. At which point in time, post-operatively, are patients seen in the out-patient clinic?

Total Hip clients: average number of WEEKS post-op

Total Knee clients: average number of WEEKS post-op

56. Do you have an established discharge criterion for total joint patients?

- Yes
- No

57. If yes, please share with us your discharge criterion for total hip/total knee patients:

58. Upon discharge from the out-patient program, do you refer any of your patients for follow-up in the community?

- Yes
- No
- Other (please specify)

59. If "yes", where do you refer patients for follow-up? (Check all that apply)

- [ ] Private practice PT
- [ ] Recreation Center
- [ ] Home and Community Care
- [ ] Other (please specify)
